# Factors Associated with Anemia in the Institutionalized Elderly

**DOI:** 10.1371/journal.pone.0162240

**Published:** 2016-09-08

**Authors:** Emanuelle Cruz da Silva, Anna Karla Carneiro Roriz, Michaela Eickemberg, Adriana Lima Mello, Elvira Barbosa Quadros Côrtes, Caroline Alves Feitosa, Jairza Maria Barreto Medeiros, Lílian Barbosa Ramos

**Affiliations:** 1 Food, Nutrition and Health Graduate Program, Federal University of Bahia (Universidade Federal da Bahia - UFBA), Salvador, Bahia, Brazil; 2 Aging-Related Research and Intervention Center, UFBA, Salvador, Bahia, Brazil; 3 Department of Nutritional Science, School of Nutrition, UFBA, Salvador, Bahia, Brazil; 4 Collective Health Graduate Program, Collective Health Institute, UFBA, Salvador, Bahia, Brazil; 5 Bahian School of Medicine and Public Health, Salvador, Bahia, Brazil; 6 Department of Medicine, Faculty of Medicine, UFBA, Salvador, Bahia, Brazil; Istituto Di Ricerche Farmacologiche Mario Negri, ITALY

## Abstract

As a common problem in long-term care facilities (LTCFs), anemia affects 25–63% of the elderly. The aim of the present study was to describe the prevalence and characteristics of anemia and its associated factors in the institutionalized elderly. The cross-sectional study was carried out with three hundred thirteen individuals aged ≥ 60 years, of both genders, living in long-term care facilities for the elderly in Salvador, Bahia, Brazil. Poisson regression (PR) with robust variance estimates was used to assess the factors related to anemia. The prevalence of anemia was 38%. Mild anemia was predominant in both genders (male: 26.8%; female: 21.1%), as normocytic and normochromic anemia, with no anisocytosis (69.75%). Anemia was associated with thinness (PR: 1.68; 95% CI: 1.04–2.72) and with moderate (PR: 1.98; 95% CI: 1.07–3.63) and total (PR: 2.61; 95% CI: 1.34–5.07) dependence in the final model. Severe dependence exhibited borderline significance (PR: 1.94; 95% CI: 1.00–3.77). The prevalence of anemia was high in the institutionalized elderly in both genders, with characteristics suggesting chronic diseases as the causal factor, and the frequency of occurrence was higher in thinness elderly with moderate to total dependence.

## Introduction

The senescence is marked by reduced hemoglobin levels, however, anemia should not be considered a natural consequence of the physiology of aging [1]. The most common causes of anemia in the elderly population are nutritional deficiencies, anemia of chronic diseases and unexplained anemia [2]. In this population, anemia has a negative impact on the health and quality of life, possibly acting as a risk factor for the development and aggravation of cardiovascular diseases and premature death, in addition to causing symptoms such as fatigue and reduced cognitive and functional capacity [3].

As a common problem in long-term care facilities (LTCFs), anemia affects 25–63% of the elderly [4]. Weight loss and protein-energy malnutrition are important etiological factors of anemia in this population [4,5], most likely due to aging-related physiological changes, reduced food intake, the presence of multiple comorbidities and insufficient nutritional care [5].

Considering anemia as an event with a high prevalence in LTCFs and its impact on the health of elderly subjects, the need to investigate the prevalence, characteristics and associated factors of anemia in this population is evident and is thus the objective of the present study.

## Materials and Methods

### Study design

The present cross-sectional study is part of a larger project titled “Multidimensional evaluation of the elderly living in long-term care facilities in Salvador, Bahia (Avaliação multidimensional dos idosos residentes em instituições de longa permanência na cidade de Salvador-BA)”, conducted by the Aging-Related Research and Intervention Center (Centro de Estudos e Intervenção na Área de Envelhecimento—CEIAE) of the School of Nutrition of the Federal University of Bahia.

### Samples

The sample of the larger study was performed in three stages. In the first stage, was identified a total of 29 LTCFs which were located in 10 Health Districts of the 12 existing in the urban area. In the second stage, the number of elderly subjects by Health District that would participate in the study was determined. This number was proportional to the total elderly population living in each Health District, thus ensuring 80% power in representing the institutionalized elderly of the city. At a significance level of 5%, this number totaled 412 elderly subjects of both genders. In the third stage, LTCFs and elderly subjects were selected by simple random sampling. The final sample information available biochemical tests was 313 elderly evaluated.

### Criteria for Eligibility

Individuals of both genders, aged 60 years and older, living in LTCFs (public, philanthropic or private) located in the urban area of Salvador, Bahia, and who agreed to participate were considered eligible to participate in the present study.

Non-eligibility criteria for the bioelectrical impedance examination included limb amputation, the presence of edema and/or ascites, the use of a cardiac defibrillator or pacemaker, and the impossibility to assess body weight [6]. Participants who could not move and/or be positioned to perform the necessary measurements were not included in the anthropometric evaluation.

### Data Collection

Data were collected from November 2012 to October 2013. The elderly participants underwent an anthropometric evaluation, bioimpedance and blood was collected to perform a complete blood count and to determine the fasting glucose and creatinine levels. A laboratory technician collected blood from participants by venipuncture after a 12-h fast to the Federal University of Bahia for laboratory analysis.

The present study was approved by the Ethics Committee of the School of Nutrition of the Federal University of Bahia under the protocol number 11/2012. Prior authorization was sought from the LTCFs, and the elderly agreed in participating in this research by signing a written informed consent using a signature or fingerprint. At the end of the study, the results from the evaluations were presented to the LTCFs using a report.

### Variables

#### Dependent Variable

A CELL-DYN Ruby hematology analyzer (Abbott Laboratories^®^, Illinois, United States) using impedance technology was used for complete blood count determination. The diagnosis and degree of anemia were established using the total blood hemoglobin levels according to the cut-off points recommended by the WHO [7].

The hematological parameters used to characterize anemia were the mean corpuscular volume (MCV), mean corpuscular hemoglobin concentration (MCHC) and red blood cell distribution width (RDW). The reference values established by the laboratory for these parameters were 80.0–99.0 fl for MCV, 31.5–35.5% for MCHC, and 11.0–14.0% for RDW.

#### Covariates

The covariates analyzed were gender, age, length of institutionalization, type of institution, body mass index (BMI), skeletal muscle index, diabetes mellitus, systemic arterial hypertension, functional capacity and renal function.

The gender, age, length of institutionalization, type of institution and use of medication were obtained from medical files.

The body mass index was calculated according to the formula suggested by the WHO [8], and analyzed according to the classification suggested by the Nutrition Screening Initiative: thinness (< 22 kg/m^2^), eutrophy (22,0–27,0 kg/m^2^), overweight (> 27 kg/m^2^) [9]. Body weight was assessed using a Plena portable digital scale (Sport model) with a maximum capacity of 150 kg and a 100-g accuracy, according to the standards determined by Jellife [10]. Height was estimated from knee height (KH) using the equations suggested by Chumlea *et al*. [11]. KH was assessed using a caliper according to the method described by Chumlea *et al*. [11].

Skeletal muscle mass was estimated using the equation described by Janssen *et al*. [12]: SM mass (kg) = [(height ^2^ ∣ R ×0.401) + (gender × 3.825) +(age × −0.071)] + 5.102; where R is resistance as measured by Biodynamics tetrapolar bioelectrical impedance analyzer (model 450), according to the technical standards and specific previous instructions described by Kyle *et al*. [6]. The skeletal muscle index was used to normalize skeletal muscle mass according to height (muscle mass (kg)/height (m^2^)) and was classified according to Janssen *et al*. [13]: adequate (man ≥ 10,76 kg/m; women ≥6,76 kg/m^2^), moderate sarcopenia (man 8,51–10,75 kg/m; women 5,76–6,75 kg/m^2^) e severe (man ≤ 8,50 kg/m^2^; women ≤ 5,75 kg/m^2^).

The capacity to perform activities of daily living was measured using the original Barthel scale [14] and the cut-off points suggested by Azeredo and Matos [15].

Fasting glucose was assessed using the Trinder reaction and a BT 3000 Plus device (Wiener lab^®^, Rosario, Argentina). Diabetes mellitus was determined by fasting glucose levels were ≥ 126 mg/dL [16] or use of oral insulin or hypoglycemic agents regularly. Hypertension was determined by the regular use of antihypertensive medication.

Renal function was evaluated by estimating the glomerular filtration rate, which was calculated from serum creatinine levels [17] using the equation described by Cockcroft and Gault. Creatinine clearance was corrected for a standard body surface area of 1.73 m^2^. Body surface area was calculated using the DuBois & Dubois formula [18]. Renal dysfunction was established at a glomerular filtration rate < 60 mL/min/1.73 m^2^ that, according to the National Kidney Foundation/Kidney Disease Outcomes Quality criteria, corresponds to stages 3 and 4 of chronic kidney disease [19]. Serum creatinine levels were assessed using a BT 3000 Plus device (Wiener lab^®^, Rosario, Argentina) and the alkaline picrate (Jaffé reaction) method.

### Statistical Analysis

Data were tested for normality using the Kolmogorov-Smirnov test for all variables. Parametric continuous variables are expressed as the mean and standard deviation, and non-parametric variables are expressed as the median and interquartile range. Categorical variables are expressed as absolute and relative frequencies. Differences in the mean values of the continuous variables with normal and non-normal distribution between the genders were assessed by Student’s *t* and Mann-Whitney *U* tests, respectively.

The correlation between hemoglobin levels and continuous covariates was determined using Spearman’s rank correlation coefficient. Pearson’s chi-squared test was used to evaluate the association between the groups with and without anemia and the remaining categorical variables.

The relationship between anemia and covariates was assessed using Poisson regression with robust error variance, estimating the prevalence ratio and its respective 95% confidence intervals. This model was used due to the possible clustering effect of the aggregation of the observation units at the facilities. The regression models were constructed from a complete regression equation using stepwise backward elimination to obtain the final reduced model.

Data were analyzed using the software Stata, version 10.0 (Stata Corp, College Station, Texas, United States), and the level of significance was set at 5% for all analyses.

## Results

**Fig 1:**
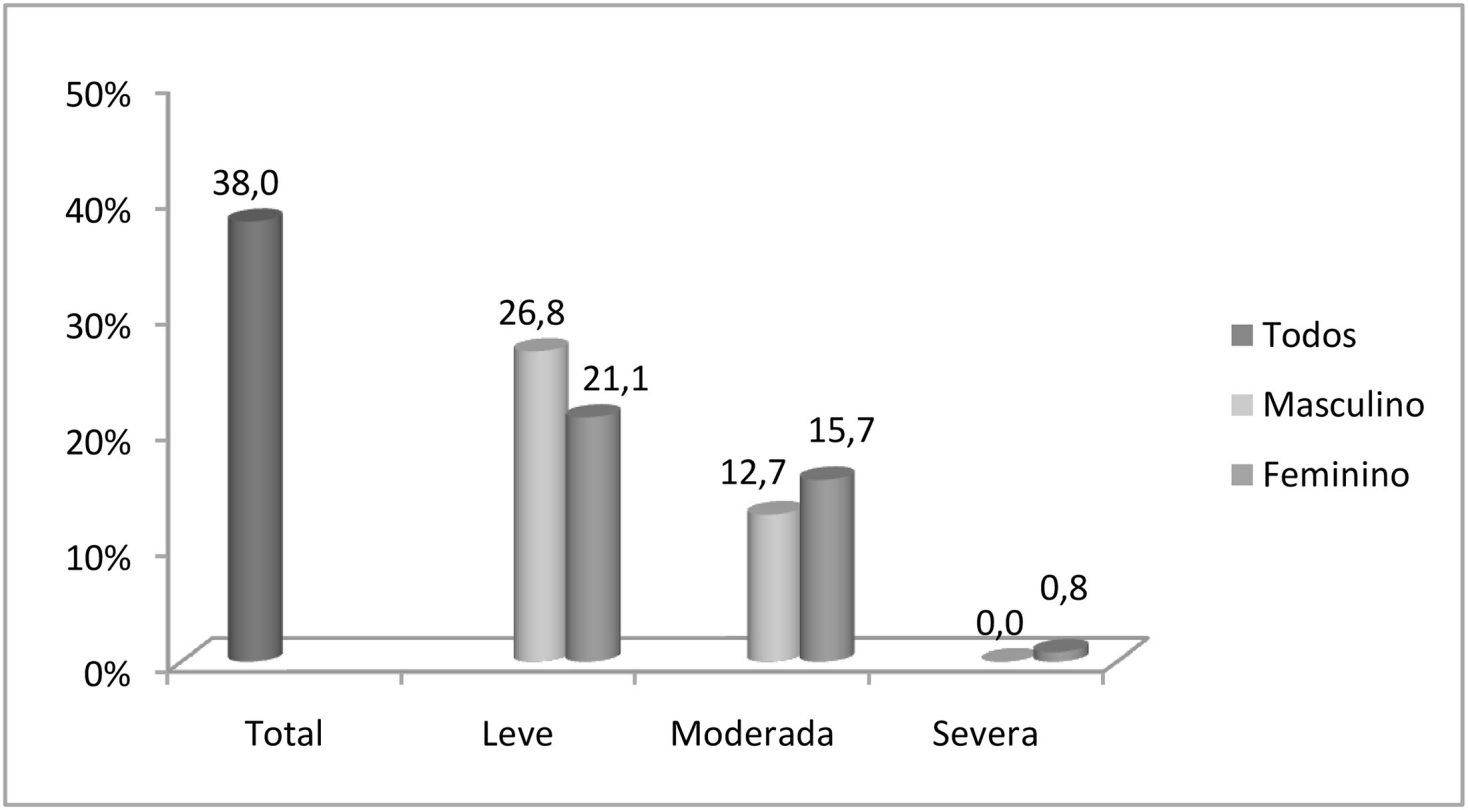
Total prevalence and degrees of anemia according to gender in the institutionalized elderly in Salvador, Bahia, Brazil.

The descriptive statistics of the participants are describe in Table 1. Anemia had an overall prevalence of 38.0% among the elderly participants (95% CI: 32.6–43.4), and mild anemia was the most prevalent form in both genders (Fig 1).

**Table 1:** Characteristics of the Institutionalized Elderly in Salvador, Bahia, Brazil according to Gender.

Variable	Women (*n* = 242)	Men (*n* = 71)	p-value
Age in years *	81.66 (9.03)	75.40 (8.82)	< .001
BMI in kg/m^2^ *	22.79 (5.53)	22.05 (4.15)	.326
GFR in mL/min/1.73 m^2^ *	45.63 (16.68)	54.20 (16.46)	< .001
Hemoglobin in g/dl *	12.25 (1.40)	13.02 (1.67)	< .001
MCV in fl *	90.19 (5.83)	87.99 (5.26)	.004
MCHC as % *	32.45 (0.88)	32.96 (0.89)	< .001
RDW as % ^†^	12.55 (11.9–13.2)	12 (11.7–12.8)	.002
Length of institutionalization in years ^†^	3 (1.33–8.41)	2.66 (0.91–5.25)	.127
SMI in kg/m^2^ ^†^	6.24 (5.57–6.98)	8.66 (8.15–9.70)	< .001
Barthel score ^†^	75 (25–95)	75 (15–95)	.565
Fasting glucose in mg/dl ^†^	86 (76–95)	82 (73.5–93)	.113

BMI, body mass index; GFR, glomerular filtration rate; MCV, mean corpuscular volume; MCHC, medium corpuscular hemoglobin concentration; RDW, red blood cell distribution width; SMI, skeletal muscle index.

* Expressed as the mean (standard deviation) and evaluated with Student’s *t* test.

^†^ Expressed as the median (interquartile range) and evaluated with Mann-Whitney *U* test.

Most of the elderly participants exhibited normochromic and normocytic anemia, with no anisocytosis. The presence of hypochromic and microcytic anemia, with and without anisocytosis, displayed similar percentages (0.84%). Hypochromia and normocytosis with no changes in the RDW occurred in 6.72% of the elderly with anemia. The portion of elderly participants with normochromic and normocytic anemia without anisocytosis was of 69.75% (Table 2).

**Table 2:** Characterization of Hematological Parameters in the Institutionalized Elderly in Salvador, Bahia, Brazil in the Presence or Absence of Anemia.

MCHC	MCV	RDW	Anemia	No anemia
			n (%)	n (%)
Hypochromia	Microcytosis	Reduced	0 (0.00)	0 (0.00)
		Normal	1 (0.84)	1 (0.52)
		Increased	1 (0.84)	0 (0.00)
	Normocytosis	Reduced	0 (0.00)	0 (0.00)
		Normal	8 (6.72)	14 (7.22)
		Increased	3 (2.52)	2 (1.03)
	Macrocytosis	Reduced	0 (0.00)	0 (0.00)
		Normal	0 (0.00)	0 (0.00)
		Increased	0 (0.00)	0 (0.00)
Normochromia	Microcytosis	Reduced	0 (0.00)	0 (0.00)
		Normal	7 (5.88)	1 (0.52)
		Increased	1 (0.84)	0 (0.00)
	Normocytosis	Reduced	5 (4.20)	13 (6.70)
		Normal	83 (69.75)	156 (80.41)
		Increased	7 (5.88)	3 (1.55)
	Normocytosis	Reduced	0 (0.00)	0 (0.00)
		Normal	3 (2.52)	4 (2.06)
		Increased	0 (0.00)	0 (0.00)

MCHC, medium corpuscular hemoglobin concentration; MCV, mean corpuscular volume; RDW, red blood cell distribution width; n, number of individuals with and without anemia; %, prevalence of individuals with and without anemia.

Specifically, anemia was observed in 37.6% of the women and 39.4% of the men, with no significant difference between the genders. It was identified high prevalence of anemia in elderly subjects aged between 60 and 69 years as well as in those aged ≥80 years, with a similar prevalence for both age ranges (39.1%), living in LTCFs for 5 to 10 years (41.7%), living in philanthropic LTCFs (41.2%), with diabetes mellitus (43.9%), with adequate skeletal muscle mass (41.5%) and systemic arterial hypertension (38.6%) and with no renal dysfunction (41.3%). Anemia was significantly associated with body mass index (*p* = 0.022) and functional capacity (*p* = 0.004). The remaining variables were not significantly associated with anemia (Table 3).

**Table 3:** Prevalence of Anemia in the Institutionalized Elderly in Salvador, Bahia, Brazil, according to Covariates.

Covariate	n/N	%	PR *	95% CI	P-value
**Gender**					
Female	91/242	37.6	1		
Male	28/71	39.4	0.95	0.68–1.32	.77
**Age Range**					
60–69 years	18/46	39.1	1		
70–79 years	31/88	35.2	0.90	0.50–1.61	.72
≥80 years	70/179	39.1	0.99	0.59–1.67	.99
**Length of institutionalization**					
<1.0 year	23/65	35.4	1		
1.0–5.0 year(s)	49/134	36.6	1.03	0.63–1.70	.90
5.1–10.0 years	20/48	41.7	1.18	0.65–2.14	.59
>10.0 years	19/53	35.0	1.01	0.55–1.86	.04
**Type of Institution**					
Private	39/109	35.7	1		
Public	14/44	31.8	0.88	0.48–1.63	.71
Philanthropic	66/160	41.2	1.15	0.77–1.71	.48
**BMI** ^†^					
Eutrophy	25/88	28.4	1		
Thinness	58/123	47.2	1.66	1.04–2.65	.03
Overweight	22/58	37.9	1.34	0.75–2.37	.32
**DM**					
No	94/256	36.7	1		
Yes	25/5	43.9	1.19	0.77–1.86	.43
**SMI**					
Adequate	17/41	41.5	1		
Moderate sarcopenia	21/62	33.9	0.82	0.43–1.55	.54
Severe sarcopenia	13/46	28.3	0.68	0.33–1.40	.30
**Functional Capacity** ^†^					
Independence	13/63	20.6	1		.26
Mild dependence	13850	32.4	1.57	0.72–3.44	.05
Moderate dependence	23/56	41.1	1.99	1.01–3.93	.04
Severe dependence	25/60	41.7	2.02	1.03–3.95	< .01
Total dependence	37/70	52.9	2.56	1.36–4.82	
**SAH**					
No	68/180	37.8	1		
Yes	51/132	38.6	0.98	0.68–1.41	.90
**Renal dysfunction**					
No	26/63	41.3	1		
Yes	76/195	39.0	0.94	0.60–1.47	.80

n, number of individuals with and without anemia; N, number of group individuals; %, Prevalence of Anemia; PR, prevalence ratio; CI, confidence interval; BMI, body mass index; DM, diabetes mellitus; SMI, skeletal muscle index; SAH, systemic arterial hypertension.

* Poisson regression model with the gross prevalence ratio for the association between anemia and other variables.

^†^ Statistical significance according to chi-squared test (BMI: *p* = 0.022; functional capacity: *p* = 0.004).

Concerning the prevalence ratio evaluation, body mass index and functional capacity were also significantly associated with anemia. The occurrence of anemia was 66% (PR: 1.66; 95% CI: 1.04–2.65) higher in elderly subjects with thinness than in eutrophic participants. Impaired functional capacity was also associated with anemia. The prevalence of anemia increased with the degree of dependence, reaching a maximum of a 156% (PR: 2.56; 95% CI: 1.36–4.82) higher prevalence of anemia in elderly subjects with total dependence than that in independent subjects (Table 3).

Despite the lack of an association between anemia and the remaining covariates, the hemoglobin levels correlated positively with skeletal muscle index (*r* = 0.169, *p* = 0.039) and glomerular filtration rate (*r* = 0.261, *p*< 0.001), and negatively with age (*r* = -0.165, *p* = 0.003).

Table 4 shows the results from the multivariate Poisson regression model, considering the possible clustering effect of the aggregation of observation units at the facilities. Thinness, as determined by body mass index, and total and severe dependence, as diagnosed using the Barthel scale, exhibited statistical significance in model 1, which included all variables. Elderly participants diagnosed with thinness exhibited a 68% (PR: 1.68; 95% CI: 1.04–2.72) higher prevalence of anemia than that in eutrophic elderly individuals, adjusting for the remaining variables of the model. Those with total and severe dependence exhibited a 212% (PR: 3.12; 95% CI: 1.14–8.52) and a 145% (PR: 2.45; 95% CI: 1.22–4.95) higher prevalence of anemia, respectively, than that in the independent elderly, adjusting for the remaining variables of the model.

**Table 4:** Poisson Regression Model with the Prevalence Adjusted for the Association between Anemia and Covariates in the Institutionalized Elderly in Salvador, Bahia, Brazil.

Covariate	Model 1 *		Model 2 ^†^	
	PRadj	95% CI	PRadj	95%CI
**Gender**				
Male	1		1	
Female	1.12	0.73–1.71	1.01	0.75–1.36
Age range				
60–69 years	1		1	
70–79 years	1.11	0.63–1.97	0.86	0.57–1.30
≥80 years	1.42	0.79–2.56	0.80	0.57–1.12
**Time of institutionalization**				
<1.0 year	1		-	-
1.0–5.0 year(s)	0.75	0. 48–1.20	-	-
5.1‒10.0 years	1.09	0.63–1.88	-	-
>10.0 years	0.85	0.40–1.84	-	-
**Type of Institution**				
Private	1		-	-
Public	0.60	0.33–1.10	-	-
Philanthropic	1.70	0.96–3.01	-	-
**BMI**				
Eutrophy	1		1	
Thinness	1.68	1.04–2.72	1.58	1.02–2.44
Overweight	1.38	0.58–3.26	1.41	0.89–2.24
**DM**				
No	1		-	-
Yes	1.26	0.79–2.03	-	-
SMI				
Adequate	1		-	-
Moderate sarcopenia	0.95	0.45–2.02	-	-
Severe sarcopenia	0.57	0.27–1.25	-	-
**Functional Capacity**				
Independence	1		1	
Mild dependence	1.94	0.62–6.10	1.58	0.67–3.72
Moderate dependence	2.04	0.88–4.76	1.98	1.07–3.63
Severe dependence	2.45	1.22–4.95	1.94	1.00–3.77
Total dependence	3.12	1.14–8.52	2.61	1.34–5.07
**SAH**				
No	1		-	-
Yes	0.79	0.47–1.33	-	-
**Renal Dysfunction**				
No	1		-	-
Yes	0.68	0.35–1.31	-	-

PRadj, adjusted prevalence ratio; CI, confidence interval; BMI, body mass index; DM, diabetes mellitus; SMI, skeletal muscle index; SAH, systemic arterial hypertension.

* Adjusted for the variables gender, age range, length of institutionalization, type of institution, BMI, DM, SMI, functional capacity, SAH and renal dysfunction.

^†^ Adjusted for the variables gender, age range, BMI and functional capacity.

In the final model, thinness maintained statistical significance but with a reduced prevalence of anemia. Additionally, moderate dependence acquired statistical significance, whereas severe dependence shifted to borderline significance. The prevalence of anemia among thinness elderly subjects was 58% higher (PR: 1.58; 95% CI: 1.02–2.44) compared with the prevalence among eutrophic individuals, adjusting for gender, age range and functional capacity. Elderly subjects with moderate, severe and total dependence exhibited a 98% (PR: 1.98; 95% CI: 1.07–3.63), 94% (PR: 1.94; 95% CI: 1.00–3.77), and 161% (PR: 2.61; 95% CI: 1.34–5.07) higher prevalence of anemia, respectively, than elderly subjects with no impairment of physical function, adjusting for gender, age range and body mass index (Table 4).

## Discussion

The prevalence of anemia found in this population (38%) was classified as moderately important at the public health level according to the parameters of WHO [7]. This prevalence was higher than what has been published by other studies on institutionalized elderly subjects [20, 21]. Studies on non-institutionalized elderly individuals have shown a lower prevalence of anemia [22, 23], supports the notion that institutionalization may be an important risk factor for the development of anemia [4].

The prevalence of anemia was similar in institutionalized men and women, corroborating with the Brazilian studies performed by Nakashima *et al*. [21] and Corona *et al*. [22]. Mild anemia was predominant in both genders, which is similar to the results obtained by Tettamanti *et al*. [24], who considered hemoglobin levels of 10.0 to 11.9 g/dL in women and 10.0 to 12.9 g/dL in men as mild anemia.

Most of the algorithms used to detect anemia in the elderly are based on RBC size, where cells are normally normocytic, due to the multifactorial origin of anemia in these individuals [25]. This finding is in agreement with the results obtained by Sgnaolin *et al*. [23] and Tettamanti *et al*. [24] who also found a predominance of normocytic anemia in the elderly.

Although there was no association between anemia and age range, blood hemoglobin concentrations decreased with advancing age, a pattern consistent with the literature, such as the studies reported by Corona *et al*. [22] and Tettamanti *et al*. [24]. This effect is possibly due to the gradual deterioration of the hematopoietic system during the aging process, thus rendering the individual at a higher risk for anemia [1]. Of note, however, the decrease in blood hemoglobin levels with increasing age of the elderly did not exhibit a dose-response effect above the lower thresholds of normality of the cut-off points adopted in the present study.

The institutionalized elderly with thinness were the most affected with anemia. Our results are in agreement with those of Tseng *et al*. [26], who observed a significant association between the occurrence of anemia and lower body mass index values in institutionalized elderly subjects.

Anemia was strongly associated with decreased functional capacity in the institutionalized elderly, a finding that is similar to the results of the studies conducted by Bosco *et al*. [27]. Both of the aforementioned studies used the Katz index to evaluate functional capacity, an instrument different from that used in the present study.

Hemoglobin levels below the threshold of normality are a common condition in individuals with chronic kidney disease [28]. While analyzing data from the Third National Health and Nutrition Examination Survey (NHANES III) to assess the association between hemoglobin levels and renal function, Astor *et al*. [29] also observed reduced hemoglobin levels with increased severity of renal dysfunction as in the present study.

The limitations of the present study include the lack of control of the use of medication and comorbidities that could affect the prevalence of anemia. A further limitation is that the nutrition of the institutionalized elderly was not considered in the analysis, although it may have affected the prevalence of anemia in this population. Finally, the inclusion of elderly subjects taking iron supplements and B-complex vitamins may have underestimated the occurrence of anemia in the present study.

The prevalence of anemia in the institutionalized elderly was high in both genders. The association of anemia with body mass index, stresses the importance of this nutritional status indicator for the identification of elderly individuals at risk for anemia.

Considering institutionalization itself as a risk factor for anemia and its negative impact on functional capacity, the importance of screening and early treatment of anemia, particularly for those who live in LTCFs, is emphasized.

## References

[1] Balducci L, Ershler WB, Krantz S. Anemia in the elderly—clinical findings and impact on health. Crit Rev Oncol Hematol. 2006; 58: 156–165. 16387511 10.1016/j.critrevonc.2005.09.003

[2] Andrès E, Federici L, Serraj K, Kaltenbach L. Update of nutrient-deficiency anemia in elderly patients. Eur J Intern Med. 2008; 19: 488–493. 10.1016/j.ejim.2008.01.016 19013375

[3] Onem Y, Terekeci H, Kucukardali Y, Sahan B, Solmazgül E, Senol MG, et al Albumin, hemoglobin, body mass index, cognitive and functional performance in elderly persons living in nursing homes. Arch Gerontol Geriatr. 2010; 50 (1): 56–59. 10.1016/j.archger.2009.01.010 19233487

[4] Morley JE. Anemia in the nursing homes: a complex issue. J Am Med Dir Assoc. 2012; 13: 191–194. 10.1016/j.jamda.2011.12.057 22261540

[5] Cereda E, Pedrolli C, Zagami A, Vanotti A, Piffer S, Opizzi A, et al Body mass index and mortality in institutionalized elderly. J Am Med Dir Assoc. 2011; 12 (3): 174–178. 10.1016/j.jamda.2010.11.013 21333917

[6] Kyle UG, Bosaeus I, De Lorenzo AD, Deurenberg P, Elia M, Manuel Gómez J, et al Bioelectrical impedance analysis—part II: utilization in clinical practice. Clin Nutr. 2004; 23 (6): 1430–1453. 15556267 10.1016/j.clnu.2004.09.012

[7] World Health Organization. Haemoglobin concentrations for the diagnosis of anaemia and assessment of severity Vitamin and Mineral Nutrition Information System. Geneva: WHO; 2011.

[8] World Health Organization. Physical status: the use and interpretation of anthropometry. Technical Report Series 854. Geneva: WHO; 1995. 8594834

[9] American Academy of Family Physicians, American Dietetic Association. A Physician's Guide to Nutrition in Chronic Disease Management for Older Adults. Washington, DC: Nutrition Screening Initiative; 2002.

[10] Jellife DB. The assessment of the nutritional status of the community (with special reference to field surveys in developing regions of the world). Monogr Ser World Health Organ. 1966; 53: 3–271. 4960818

[11] Chumlea WC, Roche AF. Steinbaugh ML. Estimating stature from knee height for persons 60 to 90 years of age. J Am Geriatr Soc. 1985; 33: 116–120. 3968366 10.1111/j.1532-5415.1985.tb02276.x

[12] Janssen I, Heymsfield SB, Baumgartner RN, Ross R. Estimation of skeletal muscle mass by bioelectrical impedance analysis. J Appl Physiol. 2000; 89: 465–471. 10926627 10.1152/jappl.2000.89.2.465

[13] Janssen I, Baumgartner RN, Ross R, Rosenberg IH, Roubenoff R. Skeletal muscle cut points associated with elevated physical disability risk in older men and women. Am J Epidemiol. 2004: 159: 413–421. 14769646 10.1093/aje/kwh058

[14] Mahoney FI, Barthel DW. Functional evaluation: The Barthel Index. Md State Med J. 1965; 14: 56–61. 14258950

[15] Azeredo Z, Matos E. Degree of dependence in stroke patients. Rev Fac Med Lisboa. 2003; 8: 199–204.

[16] Expert Committee on the Diagnosis and Classification of Diabetes Mellitus. Report of the expert committee on the diagnosis and classification of diabetes mellitus. Diabetes Care. 2002; 26 Suppl 1: S5–20. 10.2337/diacare.26.2007.s5 12502614

[17] Cockcroft DW, Gault MH. Prediction of creatinine clearance from serum creatinine. Nephron. 1976; 16: 31–41. 1244564 10.1159/000180580

[18] Du Bois D, Du Bois EF. A formula to estimate the approximate surface area if height and weight be known. 1916. Nutrition. 1989; 5: 863–871. 2520314

[19] National Kidney Foundation. K/DOQI clinical practice guidelines for chronic kidney disease: evaluation, classification, and stratification. Am J Kidney Dis. 2002; 39 Suppl 1: S1–266. 11904577

[20] Macêdo VF, Correia LO, Scoralick FM, Piazzolla LP, Macêdo DLS. Prevalence of anemia in nursing home for the aged in Brasília/DF. Rev Bras Geriatr Gerontol. 2011; 5: 214–219.

[21] Nakashima AT, de Moraes AC, Auler F, Peralta RM. Anemia prevalence and its determinants in Brazilian institutionalized elderly. Nutrition. 2012; 28: 640–643. 10.1016/j.nut.2011.09.016 22189197

[22] Corona LP, Duarte YAO, Lebrão ML. Prevalência de anemia e fatores associados em idosos: evidências do Estudo SABE. Rev Saude Publica. 2014; 48: 723–731. 25372162 10.1590/S0034-8910.2014048005039 PMC4211575

[23] Sgnaolin V, Engroff P, Ely L, Schneider RH, Schwanke CHA, Gomes I, et al Hematological parameters and prevalence of anemia among free-living elderly in south Brazil. Rev Bras Hematol Hemoter. 2013; 35 (2): 115–118. 10.5581/1516-8484.20130032 23741189 PMC3672121

[24] Tettamanti M, Lucca U, Gandini F, Recchia A, Mosconi P, Apolone G, et al Prevalence, incidence and types of mild anemia in the elderly: the "Health and Anemia" population-based study. Haematologica. 2010; 95 (11): 1849–1856. 10.3324/haematol.2010.023101 20534701 PMC2966906

[25] Andrès E, Serraj K, Federici L, Vogel T, Kaltenbach G. Anemia in elderly patients: new insight into an old disorder. Geriatr Gerontol Int. 2013; 13: 519–527. 10.1111/ggi.12017 23253055

[26] Tseng CK, Lin CH, Hsu HS, Ho CT, Huang HY, Liu CS, et al In addition to malnutrition and renal function impairment, anemia is associated with hyponatremia in the elderly. Arch Gerontol Geriatr. 2012; 55 (1): 77–81. 10.1016/j.archger.2011.06.019 21763015

[27] Bosco RM, Assis EP, Pinheiro RR, Queiroz LC, Pereira LS, Antunes CM. Anemia and functional capacity in elderly Brazilian hospitalized patients. Cad Saude Publica. 2013; 29: 1322–1332. 23843000 10.1590/s0102-311x2013000700007

[28] Stauffer ME, Tao F. Prevalence of Anemia in Chronic Kidney Disease in the United States. PLoS One. 2014; 9: e84943–e8494. 10.1371/journal.pone.0084943 24392162 PMC3879360

[29] Astor BC, Muntner P, Levin A, Eustace JA, Coresh J. Association of kidney function with anemia: the Third National Health and Nutrition Examination Survey (1988–1994). Arch Intern Med. 2002; 162: 1401–1408. 12076240 10.1001/archinte.162.12.1401

