# Correction: Factors Associated with Anemia in the Institutionalized Elderly

**DOI:** 10.1371/journal.pone.0169377

**Published:** 2016-12-22

**Authors:** Emanuelle Cruz da Silva, Anna Karla Carneiro Roriz, Michaela Eickemberg, Adriana Lima Mello, Elvira Barbosa Quadros Côrtes, Caroline Alves Feitosa, Jairza Maria Barreto Medeiros, Lílian Barbosa Ramos

Fig 1 is published in Portuguese. Please see the English version here.

**Fig 1:**
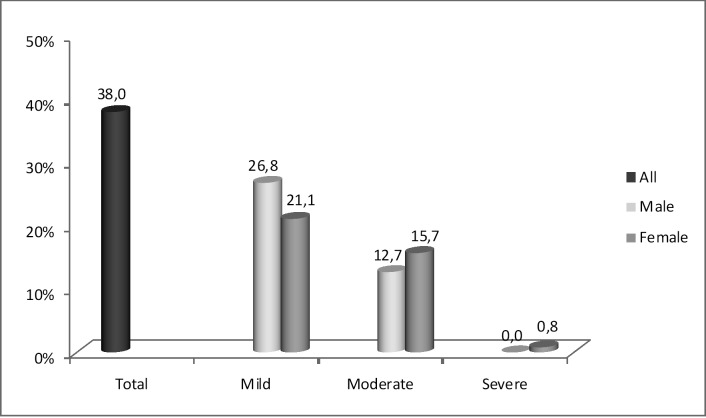
Total prevalence and degrees of anemia according to gender in the institutionalized elderly in Salvador, Bahia, Brazil.
